# Supplementary material for: HIVprotI: an integrated web based platform for prediction and design of HIV proteins inhibitors
Source: J Cheminform. 2018 Mar 9;10:12. doi: 10.1186/s13321-018-0266-y (PMC5845081; doi:10.1186/s13321-018-0266-y)
Supplement: Supplementary file 2 — Additional file 2. Source code of HIVProtI web server. [file 13321_2018_266_MOESM2_ESM.zip › HIVprotI_Source-code/hivprotI/weka-3-6-6/doc/weka/core/tokenizers/AlphabeticTokenizer.html]

AlphabeticTokenizer


---


|  |  |  |
| --- | --- | --- |
| **Overview** \| **Package** \| **Class** \| **Tree** \| **Deprecated** \| **Index** \| **Help** \| **Weka's home** | |  |
| PREV CLASS   **NEXT CLASS** | **FRAMES**    **NO FRAMES**     **All Classes** |  |
| SUMMARY: NESTED \| FIELD \| CONSTR \| METHOD | DETAIL: FIELD \| CONSTR \| METHOD |  |


---


## weka.core.tokenizers Class AlphabeticTokenizer

```
java.lang.Object
  weka.core.tokenizers.Tokenizer
      weka.core.tokenizers.AlphabeticTokenizer
```

**All Implemented Interfaces:**: java.io.Serializable, java.util.Enumeration, OptionHandler, RevisionHandler

---

``` public class AlphabeticTokenizer extends Tokenizer ```

Alphabetic string tokenizer, tokens are to be formed only from contiguous alphabetic sequences.

**Version:**
:   $Revision: 1.2 $

**Author:**
:   Asrhaf M. Kibriya (amk14@cs.waikato.ac.nz), FracPete (fracpete at waikato dot ac dot nz)

**See Also:**: Serialized Form

---

**Constructor Summary**

`AlphabeticTokenizer()`


| **Method Summary** | |
| --- | --- |
| `java.lang.String` | `getRevision()`             Returns the revision string. |
| `java.lang.String` | `globalInfo()`             Returns a string describing the stemmer |
| `boolean` | `hasMoreElements()`             returns whether there are more elements still |
| `static void` | `main(java.lang.String[] args)`             Runs the tokenizer with the given options and strings to tokenize. |
| `java.lang.Object` | `nextElement()`             returns the next element |
| `void` | `tokenize(java.lang.String s)`             Sets the string to tokenize. |

**Methods inherited from class weka.core.tokenizers.Tokenizer**

`getOptions, listOptions, runTokenizer, setOptions, tokenize`

**Methods inherited from class java.lang.Object**

`equals, getClass, hashCode, notify, notifyAll, toString, wait, wait, wait`

**Constructor Detail**

### AlphabeticTokenizer

```
public AlphabeticTokenizer()
```


**Method Detail**

### globalInfo

```
public java.lang.String globalInfo()
```

:   Returns a string describing the stemmer

    :   **Specified by:**: `globalInfo` in class `Tokenizer`
    :   **Returns:**: a description suitable for displaying in the explorer/experimenter gui

---


### hasMoreElements

```
public boolean hasMoreElements()
```

:   returns whether there are more elements still

    :   **Specified by:**: `hasMoreElements` in interface `java.util.Enumeration` **Specified by:**: `hasMoreElements` in class `Tokenizer`
    :   **Returns:**: true if there are still more elements

---


### nextElement

```
public java.lang.Object nextElement()
```

:   returns the next element

    :   **Specified by:**: `nextElement` in interface `java.util.Enumeration` **Specified by:**: `nextElement` in class `Tokenizer`
    :   **Returns:**: the next element

---


### tokenize

```
public void tokenize(java.lang.String s)
```

:   Sets the string to tokenize. Tokenization happens immediately.

    :   **Specified by:**: `tokenize` in class `Tokenizer`
    :   **Parameters:**: `s` - the string to tokenize

---


### getRevision

```
public java.lang.String getRevision()
```

:   Returns the revision string.

    :   **Returns:**: the revision

---


### main

```
public static void main(java.lang.String[] args)
```

:   Runs the tokenizer with the given options and strings to tokenize.
    The tokens are printed to stdout.

    :   **Parameters:**: `args` - the commandline options and strings to tokenize


---


|  |  |  |
| --- | --- | --- |
| **Overview** \| **Package** \| **Class** \| **Tree** \| **Deprecated** \| **Index** \| **Help** \| **Weka's home** | |  |
| PREV CLASS   **NEXT CLASS** | **FRAMES**    **NO FRAMES**     **All Classes** |  |
| SUMMARY: NESTED \| FIELD \| CONSTR \| METHOD | DETAIL: FIELD \| CONSTR \| METHOD |  |


---
